# Wrinkle reduction in forehead and glabella regions after subcutaneous forehead lift: a retrospective analysis using the wrinkle assessment scale

**DOI:** 10.3389/fsurg.2026.1776372

**Published:** 2026-03-10

**Authors:** Seok Beom Lim, Eun Se Jung, Marine Jung, In Chang Koh, Soo Yeon Lim, Wan Cheol Ryu

**Affiliations:** 1 Department of Plastic and Reconstructive Surgery, Myunggok Medical Research Center, Konyang University Hospital, University of Konyang College of Medicine, Daejeon, Republic of Korea; 2 Weill Cornell Medicine-Qatar, Doha, Qatar; 3 Sargent College of Health & Rehabilitation Sciences, Boston University, Boston, MA, United States; 4 Department of Plastic and Reconstructive Surgery, Jenith Hospital, Ulsan, Republic of Korea

**Keywords:** facial aesthetics, forehead and glabellar wrinkles, retrospective study, subcutaneous forehead, wrinkle assessment scale, wrinkles

## Abstract

**Introduction:**

Horizontal forehead and glabellar wrinkles are key indicators of upper facial aging. Various non-surgical and surgical treatments exist; however, quantitative evidence regarding their effectiveness remains lacking. Therefore, this study aimed to assess the effectiveness and safety of SFL in improving forehead and glabellar wrinkles using the validated Wrinkle Assessment Scale (WAS).

**Methods:**

This retrospective study examined the medical records of patients who underwent SFL with a pretrichial incision at a single institution between January 2015 and September 2024. Wrinkle scores of forehead and glabella were assessed using the WAS preoperatively and at the last postoperative follow-up. Comparison of the degree of score improvement between the forehead and the glabella was carried out. Inter-rater reliability was examined in a random subset of 100 patients using the intraclass correlation coefficient (ICC). Postoperative complications were also collected.

**Results:**

A total of 697 patients were included in this study. Significant reductions in forehead and glabellar wrinkle scores were observed postoperatively. The forehead and glabellar wrinkle scores improved from 2.47 ± 1.29 and 2.14 ± 0.92 preoperatively to 1.52 ± 0.96 and 1.47 ± 0.83 postoperatively, respectively. When comparing the degree of pre-to-postoperative improvement between the two regions, the mean improvement was greater in the forehead (0.95 ± 0.85) than in the glabella (0.67 ± 0.65). Inter-rater reliability demonstrated excellent agreement (ICC = 0.954 for forehead; 0.941 for glabella). Minor complications were present in a few patients; however, no significant long-term complications were observed.

**Conclusion:**

The SFL is a safe and effective procedure for wrinkle reduction in the forehead and glabellar regions. This technique demonstrated significant anti-aging benefits, with a low complication rate, presenting an effective option for managing forehead and glabellar wrinkles.

## 1 Introduction

Wrinkles play a significant role in determining perceived age, often making individuals appear older than they actually are (1, 2). Facial wrinkles on the forehead and glabella regions have a particularly strong impact on a person's overall impression and facial aesthetics. These lines result from repetitive muscular activity, progressive skin laxity, and structural soft tissue changes. To address these concerns, botulinum toxin injections, dermal fillers, laser resurfacing, microneedling, thread lifting, and surgical forehead or brow lifting procedures have been widely used to restore a more youthful appearance (3–7). However, surgical techniques may provide more substantial structural correction in selected patients (8).

Botulinum toxin remains the most commonly performed treatment for dynamic forehead lines, providing temporary muscle relaxation. However, its effect is limited in cases of deep static wrinkles and significant skin redundancy (7). Energy-based devices and resurfacing techniques may improve skin texture but do not reposition or remove redundant tissue (5). In selected patients with pronounced skin laxity or brow ptosis, surgical approaches may provide more durable structural correction.

Various forehead lift techniques have been described, including coronal, endoscopic, and pretrichial approaches (9). Among these, subcutaneous forehead lift (SFL) performed via a pretrichial incision directly addresses the superficial soft tissue envelope. By excising redundant skin and advancing the flap, SFL may mechanically reduce horizontal forehead lines while allowing simultaneous modification of the hairline and brow position.

Several studies have explored forehead lift procedures to achieve a youthful appearance; however, they mainly focused on optimizing eyebrow position and achieving proper upper face proportions (1, 10–14). While numerous proposals have been made concerning the effectiveness of forehead lifts in addressing wrinkles, quantitative studies that score and measure the extent of this improvement are lacking. Therefore, this study aimed to review the surgical SFL technique and quantitatively analyze its outcomes, including improvements in wrinkle severity assessed using the Wrinkle Assessment Scale (WAS) a validated tool widely applied in facial wrinkle studies (4, 6, 15, 16), and incidence of complications.

## 2 Materials and methods

### 2.1 Study design, participants, and ethics

This retrospective study analyzed the medical records of patients who underwent SFL via a pretrichial incision between January 2015 and September 2024 at a single institution. Patients without available photographic records, those who had previously undergone forehead lifting and/or other concurrent forehead surgeries or procedures, and those with a follow-up period of less than 6 months were excluded from the analysis. The study was approved by the Institutional Review Board (P01-202505-01-036) and performed in accordance with the ethical principles outlined in the Declaration of Helsinki. Informed consent was obtained from all patients.

### 2.2 Surgical technique

**Figure 1:**
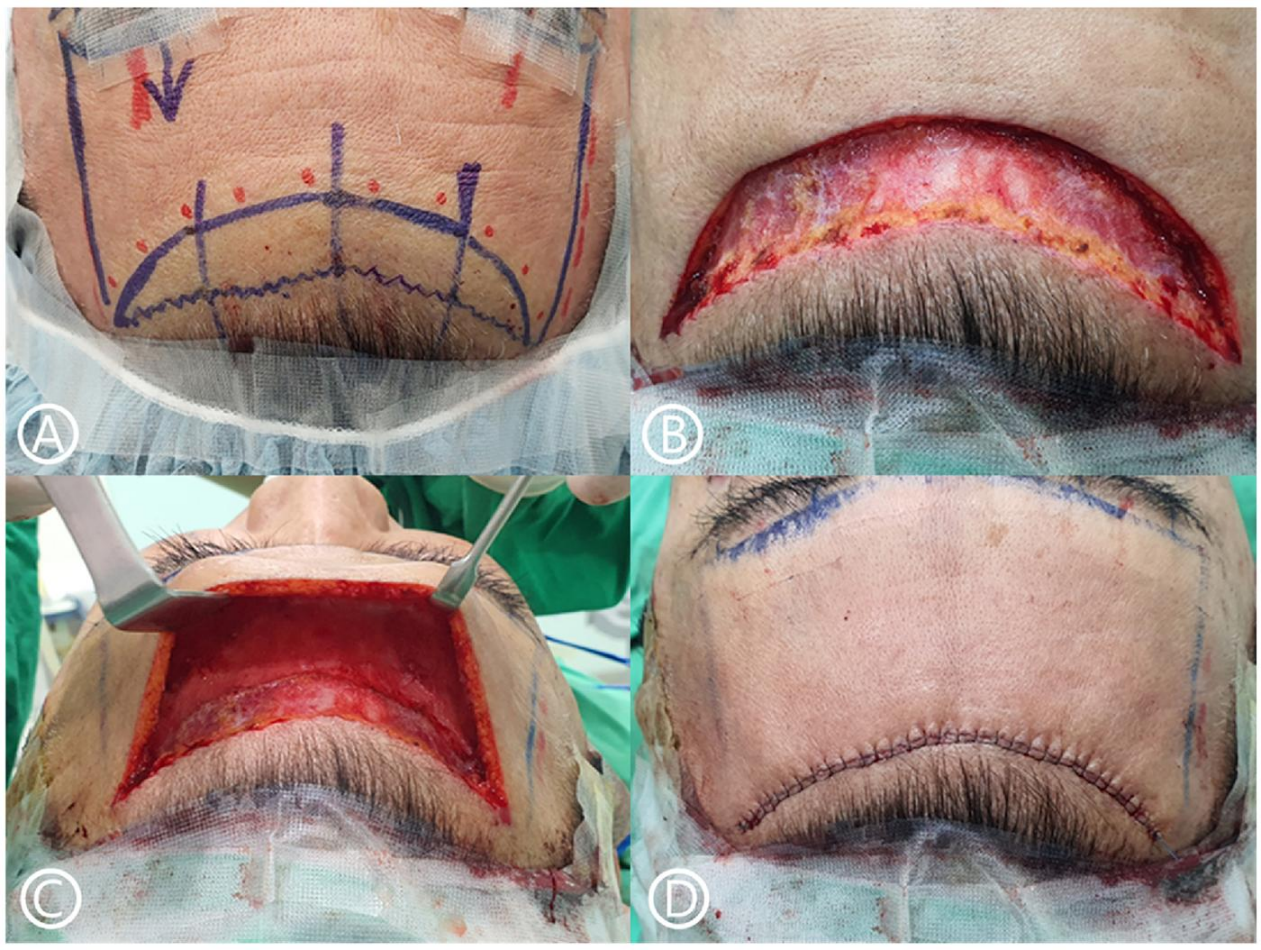
Surgical steps of subcutaneous forehead lift. **(A)** The incision line below is marked as an irregular line, while the incision line above as a convex line. **(B)** This image shows the result after skin excision. A beveled incision is used during the pretrichial incision to preserve hair follicles. **(C)** An image of the operative field after subcutaneous dissection, showing a wide field of view. **(D)** The incision is closed using PDS 3–0 and nylon 5–0 sutures.

All surgeries were performed by multiple surgeons at the same institution, using the same standardized technique (Figure 1).

### 2.3 Marking

The patient was placed in the supine position while awake, with instructions to lift the eyebrows strongly to allow proper positioning of the lower incision along the shadow lines of the forehead expression lines. The pretrichial incision line was marked in a zigzag pattern, with the shape and advancement distance adjusted according to the patient's desire and scalp laxity. An aesthetically pleasing, non-irregular convex design was applied to the lower incision line.

### 2.4 Anesthesia

Intravenous anesthesia was administered using propofol. A local anesthetic solution (10 cc of 1% lidocaine with epinephrine 1:200,000) was injected along the incision lines using a 30-gauge needle. Tumescent was injected 50–70 cc in the subcutaneous plane to minimize intraoperative bleeding and postoperative discomfort. The patient's head was slightly elevated throughout the procedures.

### 2.5 Incision

**Figure 2:**
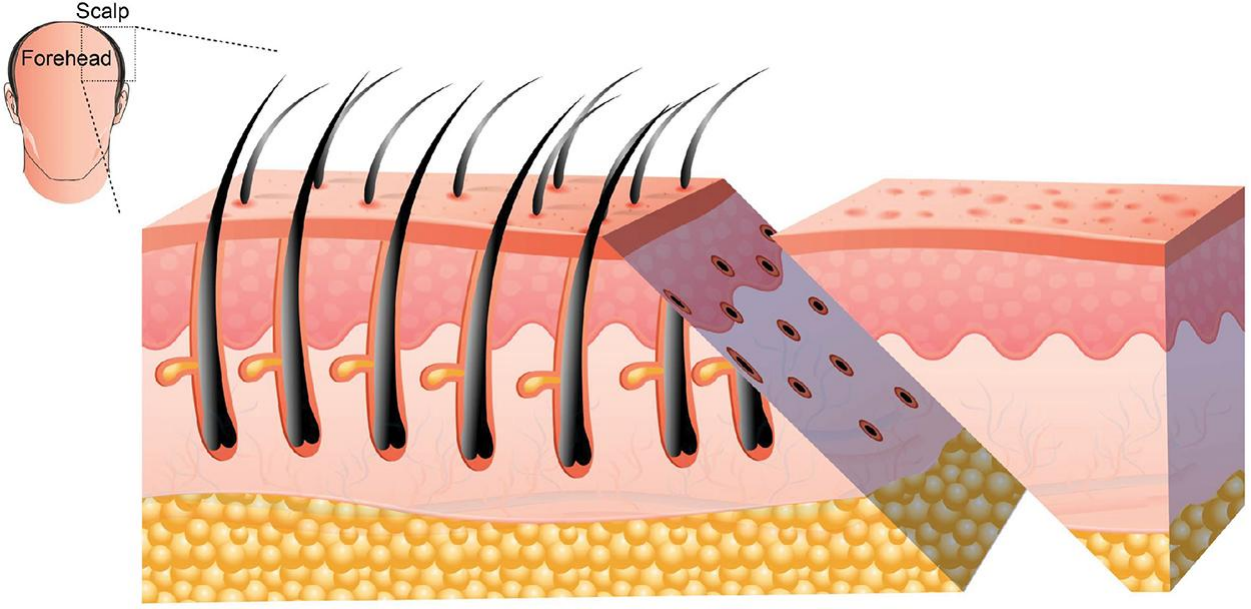
A beveled hairline incision at a 45° angle to avoid damage to hair follicles.

A pretrichial incision was made with a cutting blade beveled at approximately 45° caudally to avoid injury to the hair follicles (Figure 2).

### 2.6 Skin excision and flap elevation

The designed forehead skin was excised, and hemostasis was performed. An epinephrine-soaked gauze was placed on the cut surface of the scalp to reduce bleeding. A skin hook was used to secure the flap, and bipolar dissection elevated the flap margin. Dissection was then performed through the subcutaneous plane with a dissector or elevator to the eyebrow area, allowing for elevation of the forehead flap.

### 2.7 Forehead flap anchoring and closure

The forehead flap was advanced to the marked upper forehead line and trimmed to match the bevel of the scalp to ensure proper alignment of the scalp and forehead margins during suturing. The forehead flap was anchored to the periosteum 5 mm below the caudal margin using 3–0 PDS sutures. Adequate tissue was grasped during anchoring to prevent a relapse. Three-point anchoring was then performed.

After ensuring no tension on the flap, meticulous closure was performed using 3–0 PDS for the subcutaneous layer and 5–0 nylon for the skin. Efforts were made to ensure a neatly fitting trichophytic suture overlapping the beveled frontal and scalp incisions. A compression bandage was applied for 48 h postoperatively. Surgical drains were not used, and the skin sutures were removed on postoperative 10–12 days.

### 2.8 Outcome assessment

#### 2.8.1 The WAS

The WAS, proposed by Lemperle et al., is a tool used to quantify the severity of wrinkles on a scale of 0 to 5. The assessments were based on the patients' clinical photographs. The scale is presented in Table 1.

**Table 1:** WAS score.

WAS score	Description
0	No wrinkles
1	Just perceptible wrinkles
2	Shallow wrinkles
3	Moderately deep wrinkles
4	Deep wrinkles with well-defined edges
5	Very deep wrinkles with redundant folds

The wrinkles of the forehead and frown lines were evaluated.

WAS, wrinkle assessment scale.

The photographs were randomized and anonymized prior to evaluation. A trained evaluator (S.B.L.), who was not involved in the surgical procedures, assessed the WAS scores of the forehead and glabellar regions. The evaluator was blinded to the preoperative or postoperative status of each photograph.

To assess inter-rater reliability, a randomly selected subset of 100 patients was independently evaluated by two additional blinded observers who were not otherwise involved in this study. Intraclass correlation coefficients (ICC) were calculated using a two-way mixed-effects model with absolute agreement (average measures).

#### 2.8.2 Complications

All postoperative complications were recorded for each patient.

### 2.9 Statistical analysis

Statistical analyses were performed using IBM SPSS Statistics version 29 (IBM Corp. Armonk, N.Y.). Preoperative and final postoperative follow-up WAS scores were compared for both the forehead and glabella region. Differences in the degree of wrinkle improvement between the two regions were subsequently evaluated using a paired *t*-test. Statistical significance was set at *P* < 0.05.

## 3 Results

Between February 2015 and September 2024, 1,521 patients underwent SFL. After excluding 731 patients with a follow-up period of less than 6 months, 47 patients without adequate photographic documentation, and 46 patients who had undergone other forehead procedures or surgeries, 697 patients were ultimately included in this study (Table 2). The mean age of the patients at the time of surgery was 57.19 years, with a standard deviation of 8.15 years, and the range was 21–82 years. The mean operative time was 49 min, and operative times ranged from 20 min to 1 h and 25 min.

**Table 2:** Demographics of patients.

Characteristic	Values
No. of patients	697
Age, yrs	
Mean	57.19 ± 8.15
Range	21–82
Sex, *n* (%)	
Female	552 (79.2)
Male	145 (20.8)
Length of follow-up, months	
Mean	22.90 ± 23.11
Range	6–124
Operative time	
Mean	49 ± 15.9
Range	20–85

Data are presented as number (%).

The WAS was used to assess the severity of wrinkles in the forehead and glabellar regions preoperatively and at the last postoperative follow-up. The mean WAS score of the forehead significantly decreased from 2.47 ± 1.29 preoperatively to 1.52 ± 0.96 at the final postoperative follow-up (*P* < 0.001). Similarly, the mean WAS score of the glabella showed a significant improvement, decreasing from 2.14 ± 0.92 to 1.47 ± 0.83 (*P* < 0.001). (Table 3.).

**Table 3:** Comparison of preoperative and postoperative WAS scores for the forehead and glabella.

WAS score	Preoperative	Postoperative	*P*
Forehead	2.47 ± 1.29	1.52 ± 0.96	<0.001
Glabella	2.14 ± 0.92	1.47 ± 0.83	<0.001

**Figure 3:**
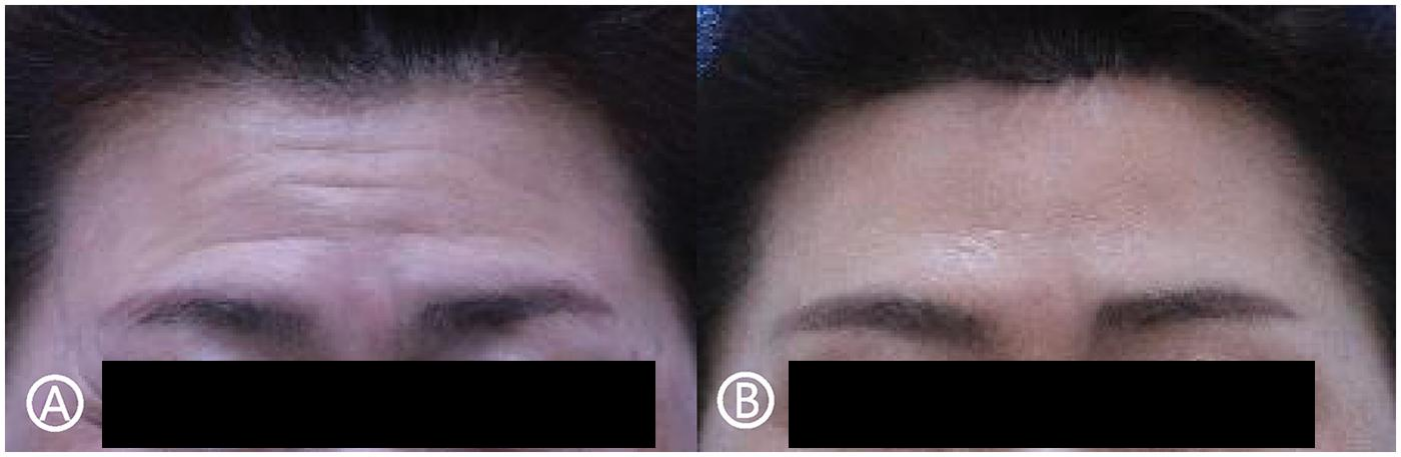
Images of a 52-year-old female patient who underwent subcutaneous forehead lift. **(A)** preoperatively, **(B)** 16 months postoperatively.

**Figure 4:**
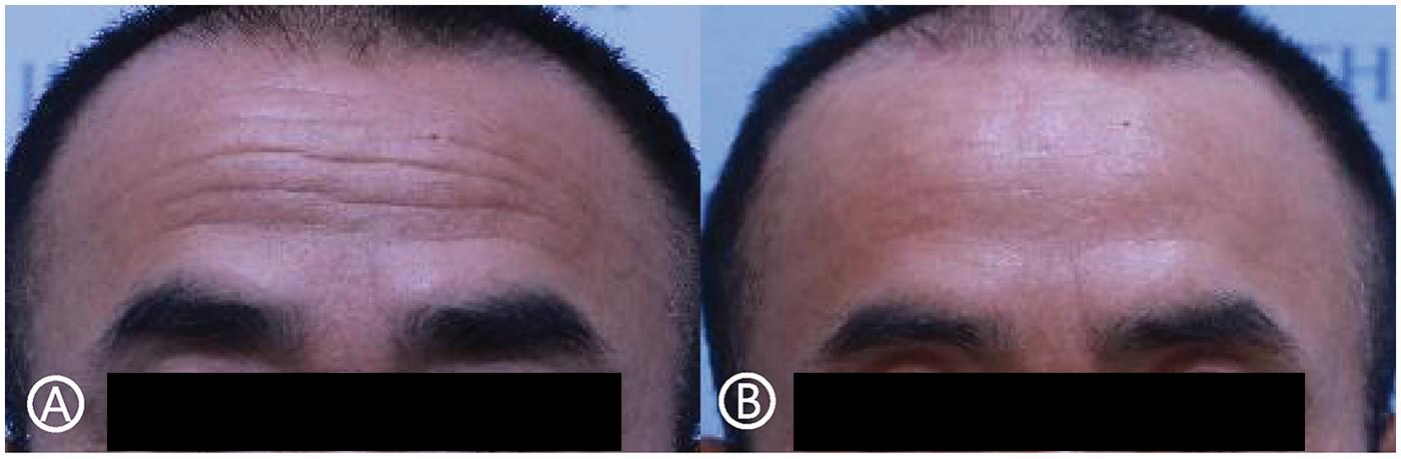
Images of a 59-year-old male patient who underwent subcutaneous forehead lift. **(A)** preoperatively, **(B)** 14 months postoperatively.

**Figure 5:**
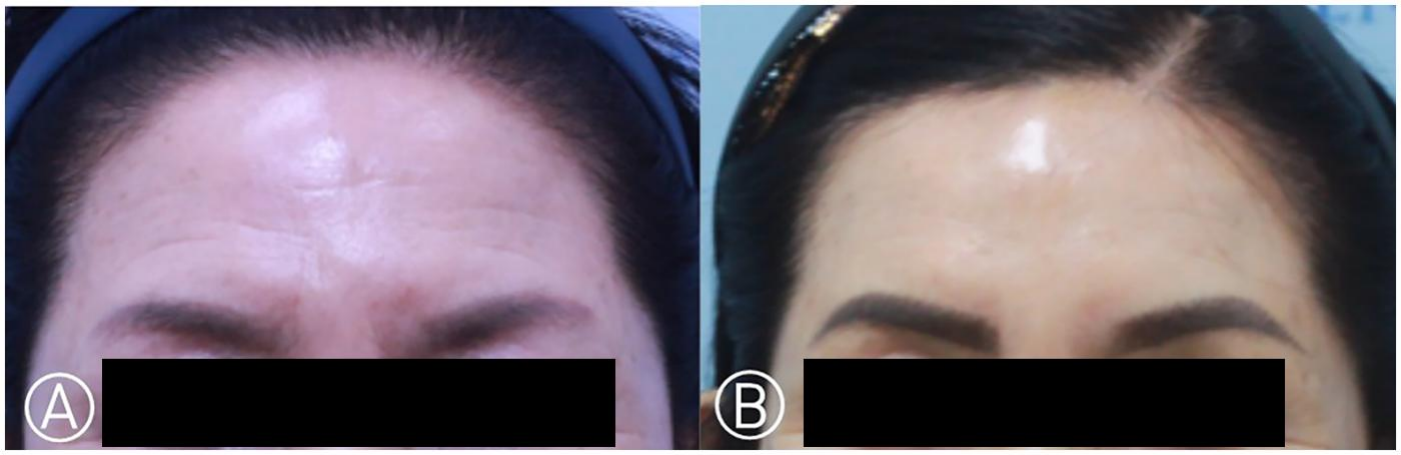
Images of a 63-year-old female patient who underwent subcutaneous forehead lift. **(A)** preoperatively, **(B)** 17 months postoperatively.

When comparing the degree of wrinkle improvement between the two regions, the forehead demonstrated a significantly greater improvement (0.95 ± 0.85) than the glabella (0.67 ± 0.65) based on paired t-test analysis (*P* < 0.001) (Table 4). Representative clinical outcomes are shown in Figures 3–5.

**Table 4:** Comparison of the degree of WAS improvement in the forehead and glabella.

Region	Forehead	Glabella	*P*
Degree of score improvement	0.95 ± 0.85	0.67 ± 0.65	<0.001

Inter-rater reliability analysis demonstrated excellent agreement. The ICC for forehead wrinkle assessment was 0.954 (95% CI, 0.942–0.964), and for glabellar wrinkle assessment was 0.941 (95% CI, 0.925–0.954) (*P* < 0.001 for both).

Of the 697 patients, 637 (91.4%) had no complications. The most common complications observed were hypertrophic scar (*n* = 32 patients, 4.6%) and prolonged numbness (*n* = 18 patients, 2.6%) (Table 5). Other complications, such as hematoma (*n* = 6 patients, 0.8%) and infection (*n* = 2, 0.3%), were also reported; however, most complications were transient and resolved with conservative management. Major complications included skin necrosis (*n* = 2, 0.3%), which resolved after dressing treatment. There have been no reports of permanent nerve injury.

**Table 5:** Complications of patients.

Complication	Number of patients
None	637 (91.4%)
Hypertrophic scar	32 (4.6%)
Prolonged numbness	18 (2.6%)
Hematoma	6 (0.8%)
Infection	2 (0.3%)
Skin necrosis	2 (0.3%)

## 4 Discussion

This study presents a large retrospective analysis of 697 patients undergoing subcutaneous forehead lift (SFL) via a pretrichial incision, demonstrating significant improvement in both forehead and glabellar wrinkles as measured by the Wrinkle Assessment Scale (WAS). The mean reduction in WAS score was 0.95 for the forehead and 0.67 for the glabella, both statistically significant (*P* < 0.001). Inter-rater reliability analysis performed on a random sample of 100 patients showed excellent agreement (ICC = 0.954 for forehead and 0.941 for glabella), supporting the reproducibility of the scoring system. The overall complication rate was low, and most adverse events were minor and self-limited.

Horizontal forehead lines represent one of the most visible markers of upper facial aging. Current treatment modalities include botulinum toxin injections, dermal fillers, laser resurfacing, microneedling, thread lifting, and surgical brow or forehead lifting procedures (3, 6, 7, 16, 17). Among these, botulinum toxin remains the most commonly performed treatment, providing temporary relaxation of the frontalis muscle and softening dynamic wrinkles for approximately 4–6 months. However, repeated treatments are required to maintain results, and its immediate effect on deep static wrinkles or redundant skin is limited (18, 19). Laser resurfacing and microneedling can improve skin texture but do not reposition tissue or address significant skin redundancy (4, 16).

In contrast, SFL directly excises redundant skin and mechanically repositions the forehead soft tissue envelope. By exclusively manipulating the subcutaneous layer, the procedure avoids deep muscle dissection, reducing the risk of damage to the deep branch of supraorbital nerves. This may enhance procedural safety and make the technique accessible to a wider range of surgeons (9). Although the superficial branch of the supraorbital nerve may be cut during skin excision, it typically recovers within 2–3 months (20). The greater improvement observed in the forehead compared with the glabella in this study may reflect anatomical differences. Forehead wrinkles are largely influenced by frontalis muscle activity and skin redundancy, which can be mechanically reduced through flap advancement. In contrast, glabellar lines are strongly affected by corrugator and procerus muscle activity, which are not directly modified in SFL unless additional muscle procedures are performed.

SFL typically involves longer incisions and greater tissue dissection, leading to longer recovery times and scarring (21). However, fewer individuals expressed dissatisfaction with their scars than anticipated (14). Although hypertrophic scars were observed in 32 patients in this study, one patient required scar revision surgery. While not addressed in this study, the pretrichial incision approach allows concomitant procedures, such as mass excision, that would otherwise result in a central forehead scar, by repositioning the scar to a less noticeable location. Furthermore, SFL enables control of hairlines and forehead length through modification of the incision line. This feature may serve as an alternative to hair transplantation for patients experiencing hairline recession.

It is important to emphasize that SFL should not be considered a universal solution for all patients presenting with forehead wrinkles. Appropriate patient selection is essential. Candidates who may benefit most from SFL include those with pronounced static horizontal wrinkles, significant skin redundancy, brow ptosis requiring repositioning, or a high hairline that can be concurrently corrected (1, 14, 21, 22). In contrast, patients with primarily dynamic wrinkles and minimal skin excess may achieve satisfactory results with less invasive modalities such as botulinum toxin (7, 17). Therefore, SFL should be viewed as one option within a broader therapeutic spectrum rather than a replacement for non-surgical treatments.

Compared with endoscopic forehead lifting techniques, SFL offers a different surgical philosophy. While endoscopic approaches operate in deeper planes and aim primarily at brow repositioning, SFL allows direct visualization of the subcutaneous layer and precise skin excision (23–25). However, given the absence of comparative data in this study, no conclusions regarding superiority over other techniques can be drawn. Future prospective comparative studies would be necessary to determine relative advantages in specific patient populations.

The low rate of adverse events observed in this cohort may be attributed to standardized surgical technique, careful beveling of the pretrichial incision to preserve hair follicles (26, 27), meticulous flap handling, and tension-free closure. Hypertrophic scarring occurred in 4.6% of patients, with one case requiring revision. Prolonged numbness was observed in 2.6% of patients and resolved over time, with no cases of permanent nerve injury reported. Nevertheless, the exclusion of patients with follow-up shorter than six months may have led to underestimation of late complications, and this represents a potential source of selection bias.

The WAS was used to assess the severity of forehead and glabellar wrinkles (15). This scale offers a more detailed assessment of wrinkle severity than other commonly used scales, such as the Fitzpatrick scale, which only offers a range from 1 to 3 points, limiting its ability to capture the full spectrum of wrinkle severity (3). Additionally, while the Glogau scale is useful for evaluating the overall degree of facial aging, it does not account for the motion and dynamic nature of wrinkles, making it less applicable to this study (28). The WAS allows for a comprehensive and accurate evaluation of wrinkle severity and has been widely validated in aesthetic procedures, making it an ideal tool for assessing the effects of SFL.

This study has some limitations. First, the retrospective design of the study introduces potential bias. As it relies on existing clinical records and many patients had a follow-up period of less than 6 months, a substantial proportion of patients were excluded due to insufficient follow-up, which may influence generalizability. Second, patient-reported outcomes such as FACE-Q were not available, limiting assessment of subjective satisfaction (29). Future prospective studies incorporating validated patient-reported outcome measures and longer standardized follow-up intervals would further strengthen the evidence base. Finally, the scoring process can involve subjective assessments. Although the evaluator was blinded and inter-rater reliability was excellent, photo-based scoring remains partially subjective. More objective evaluation methods, such as 3D scanning, could provide a more impartial assessment of outcomes. Despite these limitations, the strengths of this study include the use of a validated wrinkle assessment scale and a large sample size, providing robust evidence for the effectiveness of SFL and representing one of the largest quantitative datasets evaluating wrinkle reduction after SFL. By systematically applying a validated wrinkle scale and confirming high inter-rater reliability, this study contributes objective evidence supporting SFL as a safe and effective surgical option for selected patients with upper facial aging.

## 5 Conclusion

In this large retrospective cohort study, subcutaneous forehead lift via a pretrichial incision was associated with significant improvement in both forehead and glabellar wrinkles as measured by the Wrinkle Assessment Scale. Inter-rater reliability analysis demonstrated excellent agreement, supporting the objectivity of the photographic assessment. The overall complication rate was low, and most adverse events were minor and self-limited.

Although the absence of a control group and the retrospective design limit definitive comparative conclusions, the present findings suggest that SFL is a safe and effective surgical option for selected patients seeking structural improvement of upper facial wrinkles.

Prospective controlled studies are warranted to further validate these findings and to compare outcomes with alternative surgical and non-surgical approaches.

## Data Availability

The raw data supporting the conclusions of this article will be made available by the authors, without undue reservation.
